# Tap dancers in the wild: field observations of multimodal courtship displays in socially monogamous songbirds

**DOI:** 10.1007/s00114-020-01686-x

**Published:** 2020-07-19

**Authors:** Nao Ota

**Affiliations:** 1 Department of Behavioural Neurobiology, Max Planck Institute for Ornithology, Seewiesen, Germany; 2 JSPS Overseas Research Fellow, Japan Society for the Promotion of Science, Tokyo, Japan

**Keywords:** Dance, Multimodal communication, Mutual courtship display, Biotremology, Sonations, Estrildidae

## Abstract

Multimodal signaling systems are shaped not only by a signaler’s physical abilities but also by external factors such as the position of signal receivers and the properties of the medium through which the signals are transmitted. To fully understand the evolution and function of multimodal communication, it is essential to investigate the behavior in the wild. Here, I present evidence that socially monogamous songbirds perform complex courtship displays that can produce multimodal and multicomponent signals in wild conditions. Cordon-bleus (*Uraeginthus* spp.) are socially monogamous songbirds from East Africa. Both sexes of cordon-bleus perform multimodal courtship displays by holding a piece of nest material, bobbing up and down, and singing. My previous laboratory study using high-speed video cameras revealed that courtship bobbing includes multiple rapid steps similar to human tap-dancing, which presumably contributes to producing non-vocal sounds and/or vibrations in addition to visual signals. As a result of field observation and behavioral analysis, I found that wild cordon-bleus perform tap-dance like displays just as captive cordon-bleus. I also observed that wild cordon-bleus produced non-vocal sounds and shook branches during courtship, which can contribute to multimodal signal production (i.e., visual, acoustic, and vibrational signals). My findings imply that the courtship displays of cordon-bleus are an ideal candidate for investigating the role and function of multimodal communication in animals, and demonstrate the importance of further quantitative studies in both laboratory and field.

**Electronic supplementary material:**

The online version of this article (10.1007/s00114-020-01686-x) contains supplementary material, which is available to authorized users.

## Introduction

Multimodality is one of the factors that make animal communication complex. Multimodal signaling can contribute to enhancing the accuracy of signal perception under noisy conditions (backup signal hypothesis) and/or provide multiple messages indicating several different qualities of the sender (multiple message hypothesis, Johnstone 1996). These hypotheses work under the premise that the signals can be efficiently transmitted via appropriate mediums in wild environments (e.g., air, water and substrate). As well as the physical abilities and constraints of signalers, environmental and social conditions also have a great influence on multimodal signaling strategies and their efficacy (reviewed in Partan 2017). To obtain a comprehensive understanding of a multimodal signal and its functions, knowing the wild behavior of an animal and its surrounding environments are as important as conducting well-controlled experiments in the laboratory.

Bird courtship displays are a good example of multimodal signals which can involve the coordination of vocalizations and body movements (Cooper and Goller 2004; Dalziell et al. 2013; Ullrich et al. 2016; Miles and Fuxjager 2018). Previous reviews have provided several hypotheses and frameworks for understanding multimodal communication systems (Johnstone 1996; Hebets and Papaj 2005). However, relatively little experimental work exists on multimodal communication during courtship displays (Mitoyen et al. 2019). Moreover, little attention has been paid to modalities and signal components that can be produced by body movements such as vibrations and non-vocal sounds in the context of courtship in birds (but see Soma and Mori 2015; Hogan and Stoddard 2018). Investigating how multimodal and multicomponent signals interact and work in bird courtship displays would yield novel insights into the evolution, functions, and complexity of animal communication.

**Fig. 1:**
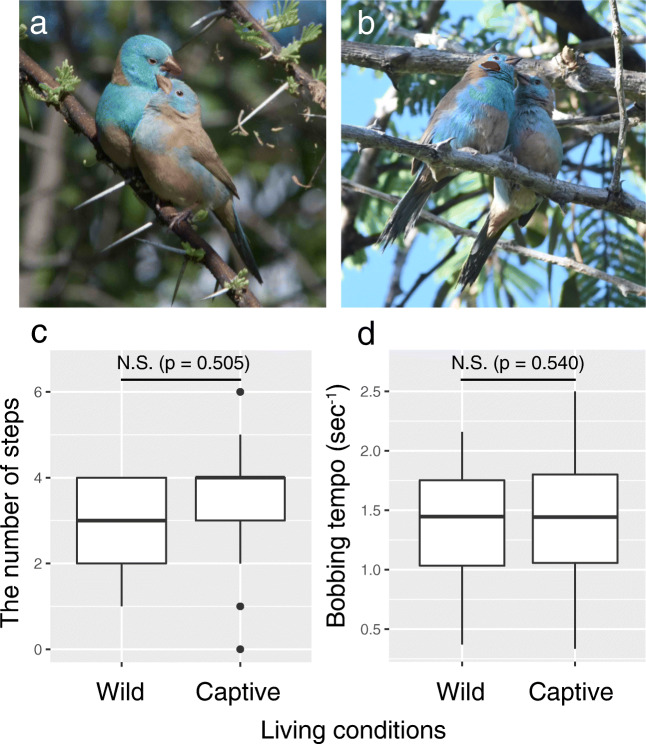
A male (left) and a female (right) of **a** blue-capped cordon-bleus (*Uraeginthus cyanocephalus*) and **b** red-cheeked cordon-bleus (*Uraeginthus bengalus*) perching in trees. The comparisons of **c** the number of steps per bobbing and **d** bobbing tempo (sec^−1^) between wild and captive male blue-capped cordon-bleus. Box plots show median and quartiles and the whiskers include the range of values within 1.5 times the interquartile range. Circles indicate outliers

In the blue-capped cordon-bleu (*Uraeginthus cyanocephalus*; Fig. 1a), a socially monogamous songbird, both male and female, repeatedly bob up and down and sing songs while holding a piece of nest material in the beak during courtship (Goodwin 1982; Ota et al. 2015, 2018). Using a high-speed camera, my previous laboratory study revealed that both male and female cordon-bleus rapidly stamp their feet several times in one bobbing (Ota et al. 2015). This tap-dance-like display generates distinct non-vocal sounds (Ota et al. 2017) and probably also produces vibrations through the bird’s perch. However, the previous studies of cordon-bleus have only been conducted in laboratory conditions and their wild courtship behavior is completely unknown.

As a first step toward understanding multimodal courtship communication in cordon-bleus under wild conditions, I conducted video recordings of blue-capped cordon-bleu courtship displays and investigated if the tap-dancing display can be observed in the field. I expected that wild cordon-bleus of both sexes would perform tap-dance-like displays in the same manner as in the laboratory. I additionally made recordings of red-cheeked cordon-bleus (*Uraeginthus bengalus*, Fig. 1b), which are also known to perform tap-dance-like displays (Ota et al. 2015), and tested if the multimodal courtship display contributes to species recognition. I also discuss the possibility of multimodal signal production using dancing displays in wild cordon-bleus and its functions by describing the results of the field observations.

## Materials and methods

I conducted field observations in Tanzania during the rainy season from March to May in 2019. Observations were made at this time of the year because cordon-bleus are opportunistic breeders and generally start to breed during this time (Goodwin 1982; see also experimental materials and procedures in Online Resource 2). Normal-speed (30 frames per second) and high-speed (240 frames per second) cameras were used to record cordon-bleu courtship displays.

Using high-speed videos, I confirmed that wild cordon-bleus perform tap-dance-like displays as observed in captive birds (Ota et al. 2015; see Video 1 included in Online Resource 1). Based on normal-speed and high-speed videos, I quantified two parameters of dance performance (the number of steps in one bobbing and bobbing tempo; Ota et al. 2015) and used them as response variables. Using the data of captive male blue-capped cordon-bleus quantified in the previous study (Ota et al. 2015) and wild male blue-capped cordon-bleus (this study), I compared the dance performances between captive and wild birds. I also compared the bobbing tempo between wild male blue-capped and wild male red-cheeked cordon-bleus to determine the species difference. I used a generalized linear mixed-effect model to analyze the number of steps and a linear mixed-effect model to analyze bobbing tempo. The number of steps in one bobbing was analyzed with a Poisson distribution and bobbing tempo was analyzed with a Gaussian distribution (Ota et al. 2015, Online resource 2). In all analyses, I considered bird ID as a random effect to control for non-independence of data. All statistical analyses were performed using R 3.4.4 (R core team 2018) with nlme (Pinheiro et al. 2020) and lme4 (Bates et al. 2015) packages.

To examine the possibility of the production of non-vocal sounds and vibrations during courtship, I examined if the non-vocal sounds were audible on the recordings as is observed in captive birds (Ota et al. 2017) and if the branches were shaken during courtship. I also checked the environmental conditions, the presence, and the sex of a signal receiver on the same substrate based on the video recordings. The detailed observational perspectives and analyses are described in the Supplementary materials (Online Resource 2).

## Results

I succeeded in filming the tap-dance like movements of five male blue-capped cordon-bleus with a high-speed camera (Video 1 in Online Resource 1). I also filmed the dance display using a normal-speed camera (29 male blue-capped cordon-bleus, four male and one female red-cheeked cordon-bleus). On average, wild male blue-capped cordon-bleus performed 2.8 steps per one bobbing action (range 1–4; Fig. 1c). The average bobbing tempo was 1.40/s. These values were close to the dance performances of this species under captive conditions (average 3.17 steps per one bobbing and 1.39 bobs/s; Ota et al. 2015). There were no significant differences in the dance performances of captive and wild male blue-capped cordon-bleus (Fig. 1c, d; Table 1). There was also no significant difference in bobbing tempo between blue-capped and red-cheeked cordon-bleus (Fig. S1; Table S1 in Online Resource 2).

**Table 1:** Results of a generalized linear mixed-effect model and a linear mixed-effect model conducted to test the effects of living conditions (captive or wild) on dance performances in male blue-capped cordon-bleus (*Uraeginthus cyanocephalus*). Bird ID was included as a random effect in both models. Estimated values for effects that contained “living conditions” term are for wild individuals

Response variables	Fixed effect	Coefficient	SE	z/t-value	*P* value	Distributions
The number of steps in one bobbing	Living conditions	− 0.102	0.153	*z* = − 0.667	0.505	Poisson
Bobbing tempo	Living conditions	− 0.061	0.097	*t* = 0.621	0.540	Gaussian

Both blue-capped and red-cheeked cordon-bleus always performed dance displays on the branches of trees (*Acacia* spp. and *Faidherbia albida*) holding various types of items in the beak (Fig. S2 in Online Resource 2). As far as I observed, cordon-bleus never performed the courtship display in the same place twice. Non-vocal sounds produced during dance displays were audible and apparent on the spectrograms from two normal-speed videos of male blue-capped cordon-bleus (Fig. S3 in Online Resource 2). I also observed that the branch of a tree was often shaken by tap-dancing displays in the videos of male blue-capped cordon-bleus (17 of 34 videos, Video 1 in Online Resource 1).

I observed that the female signal receiver perched on the same substrate in the several recording videos of males (four of 34 videos in blue-capped, two of four videos in red-cheeked cordon-bleus). I never observed song nor dance duetting behavior between a male and a female cordon-bleu.

## Discussion

The field observations in this study revealed that wild cordon-bleus exhibit tap-dance-like displays during courtship communication. As predicted, wild cordon-bleus performed rapid dance displays in the same manner as captive birds (Table 1; Video 1 in Online Resource 1). Another similarity observed between wild and captive individuals was that they both performed dance displays on branches but never on the ground (Ota et al. 2015; Video 1 in Online Resource 1).

As my data is descriptive it is too early to conclude the functions and efficacies of multimodal signals from this study. However, my field observations strongly imply that cordon-bleu dance displays can produce multimodal and multicomponent signals including non-vocal sounds and vibrations under wild conditions. I observed that some signal receivers perched on the same substrate with signal senders, and in those cases, they seemed quite possible to perceive the non-vocal sounds and vibrations. I also found that the dance displays resulted in the shaking of several branches of the tree in which the bird performed (Video 1 in Online Resource 1). Since the movements were clearly different from those caused by the wind, shaking a branch by dancing might also function as a visual signal in addition to the dance movements and items held during the display (Fig. S2 in Online Resource 2). This possibility has been overlooked in studies under laboratory conditions.

Presumably, the multimodal signals produced during cordon-bleu courtship displays can have several functions. The multimodal signals comprised of visual elements with sounds and vibrations which could function as backup signals to ensure appropriate signal transmission under fluctuating environmental conditions. It is also possible that multimodal signals may encode multiple messages directed toward multiple signal receivers (Johnstone 1996). Since the species difference in the dance performance was not detected in the current study (Fig. S1; Table S1 in Online Resource 2), the dance display might function as conveying information such as physical abilities and motivations rather than the species. More detailed analyses are required to elucidate the information encoded in the multimodal courtship displays of cordon-bleus.

An important next step following this study is to quantitatively test how multimodal and multicomponent courtship signals affect the responses of signal receivers in cordon-bleus as well as their future breeding success. My previous (Ota et al. 2015) and current studies emphasize that both field and laboratory approaches are useful for understanding multimodal signaling behavior in cordon-bleus. While laboratory experiments would enable us to conduct detailed behavioral experiments under well-controlled conditions, the signal efficacy (e.g., the effective range of sound/vibrational amplitude) and actual mating processes via courtship display should be examined in the field.

Another question that will deserve further attention is why female courtship displays were hardly observed in the wild, considering that captive female cordon-bleus exhibit courtship displays like males (Goodwin 1982; Geberzahn and Gahr 2011; Ota et al. 2015). A likely explanation is that my observations occurred during the middle or end of the cordon-bleu breeding season. Potential sex differences in the timings of courtship displays due to the sex differences in reproductive constraints and extra-pair mating strategies (Tobias et al. 2012) may, therefore, have affected the number of female displays observed in this study.

In conclusion, I have shown for the first time in wild species that cordon-bleus perform rapid “tap-dance”-like display. Though not conclusive due to the lack of quantitative evaluation of signal efficacy, the results point to the potential of multimodal signaling behavior overlooked in previous songbird communication studies.

## Electronic supplementary material

ESM 1 (PDF 508 kb)

Video 1 (MP4 4162 kb)
