# Supplementary material for: Tap dancers in the wild: field observations of multimodal courtship displays in socially monogamous songbirds
Source: Naturwissenschaften. 2020 Jul 19;107(4):30. doi: 10.1007/s00114-020-01686-x (PMC7369261; doi:10.1007/s00114-020-01686-x)
Supplement: Supplementary file 1 — (PDF 508 kb) [file 114_2020_1686_MOESM1_ESM.pdf]

Supplementary materials

Tap dancers in the wild: field observations of multimodal courtship displays in socially monogamous songbirds

Nao Ota [1,2]

[1] *Max Planck Institute for Ornithology, Department of Behavioural Neurobiology, Seewiesen, Germany*

[2] *JSPS Overseas Research Fellow, Japan Society for the Promotion of Science, Tokyo, Japan*

nao.ota1@gmail.com
ORCiD: 0000-0001-8443-1630

Experimental materials and procedures

The blue-capped cordon-bleu (*Uraeginthus cyanocephalus*) and the red-cheeked cordon-bleu (*Uraeginthus bengalus*) have sexually dimorphic plumage, and males have blue-caps in blue-capped cordon-bleus and red-cheeks in red-cheeked cordon-bleus respectively (Goodwin 1982). Blue-capped cordon-bleus also have brighter blue plumage and redder beaks than red-cheeked cordon-bleus (Goodwin 1982). The two species and the sexes can, therefore, be easily recognized and distinguished. Although it is possible that other Estrildid finches also perform tap-dance like displays, I conducted field observations focusing on two species since they are the only Estrildid finches so far reported as 'tap-dancing birds' (Ota et al. 2015).

A field assistant and I conducted field observations in two study sites: the Mbeya region (8°43'S 34°04E, for 24 days between 9/3/2019 to 5/4/2019) and the Iringa region (7°48'S 35°05E, for 22 days between 7/4/2019 to 4/5/2019) of Tanzania. The habitat in these regions is arid with bushes and trees. I determined the place and season based on descriptions in the literature (Goodwin 1982), the local fieldworker's opinions and their Bird Atlas (Baker and Baker 2016). I confirmed the report that cordon-bleus stay and breed at the above study sites during the rainy season (Baker and Baker 2016).

An ad-lib sampling method (Altmann 1974) was adopted since cordon-bleus are moderately gregarious, non-territorial birds and the location and timing of their dancing displays are not well known (Goodwin 1982). The field observations were carried out in the morning from 6:30 to 12:00 because in the afternoon, birds were hard to observe and produced little song (personal observation). A field assistant and I walked around the study site and, as soon as we observed a cordon-bleu courtship dance display, we attempted to film their behavior using normal speed (GZ-E355, JVC KENWOOD Corporation, Japan; 30 frames per second) or high-speed (GC-P100, JVC KENWOOD Corporation, Japan; 240 frames per second) cameras. The focal individuals were selected based on the bobbing movements, singing activity and/or the presence of a piece of nest material in their beaks, as those are typical courtship components of Estrildid finches including cordon-bleus (Goodwin 1982; Soma 2018). When I missed a chance to film a courtship display, I made a record of the species, sex and what they were holding in their beaks during courtship (Fig. S2).

I quantified the number of steps in one bobbing and bobbing tempo following the methods used in the previous studies (Ota et al. 2015). The number of steps in one bobbing was counted based on 5 high-speed videos in male blue-capped cordon-bleus. The number of steps was counted from an average of 10.4 ± SD 7.889 bobbings (range 1–24) per individual. Bobbing tempo was calculated using both normal-speed and high-speed videos. I defined one dance bout as a sequence of bobbings with less than 3-second intervals (Ota 2017). I quantified bobbing tempo from 21 of 34 videos in blue-capped cordon-bleu males, three of four videos and one of one video in red-cheeked cordon-bleu males and females respectively. Bobbing was calculated from an average of 3.476 ± SD 3.333 dance bouts (range 1–16) per individual in blue-capped cordon-bleus and an average of 3.25 ± SD 2.861 dance bouts (range 1–8) in red-cheeked cordon-bleus.

I excluded some videos from the analysis because it was hard to observe the dance movements in the videos due to camera shake and leaves overlapping the frame. The dance performances of seven captive male blue-capped cordon-bleus from the previous study (Ota et al. 2015) were used for the comparison of the dance performances of wild and captive male blue-capped cordon-bleus (the number of steps in one bobbing: average 359.429 ± SD 223.549 data points per individual, range 68–837; bobbing tempo: average 53.429 ± SD 46.161 data points per individual, range 9–159).

Using data from observations of dance performances in wild and captive male blue-capped cordon-bleus, I examined the effect of living conditions (captive or wild) on dance performance. To examine species differences, I compared the bobbing tempo between wild blue-capped and wild red-cheeked cordon-bleus. As the number of steps in one bobbing is discrete values (count data), it was analyzed with a Poisson distribution as with the previous study (Ota et al. 2015). Bobbing tempo was analyzed with a Gaussian distribution after confirming that the distribution did not deviate from normality (Kolmogorov Smirnov test: $p > 0.125$). In all analyses, I considered bird ID as a random effect to control for non-independence of data. Each of the birds in the 29 video data (five high-speed and 21 normal-speed videos in blue-capped cordon-bleus, four normal-speed videos in red-cheeked cordon-bleus) was treated as a different individual (i.e., each individual in a single video has a different bird ID). I did this because I could not detect if the individuals are identical between the videos, but I

assume that the recorded individuals are not identical considering that an adequate number of cordon-bleus were observed in the field (personal observation).

To investigate if the cordon-bleu dance display produces non-vocal sounds and vibrations as previously reported in captive individuals (Ota et al. 2015, 2017), behavioral and acoustic analyses were carried out using video data. I examined whether I could observe the branch being shaken according to the dance movements of signal senders in both normal-speed and high-speed video recordings. I also tested if non-vocal sounds produced during the dance display were audible and also confirmed them on spectrograms. The non-vocal sounds can be easily distinguished from vocalization because it is harsher than song syllables and is emitted in synchrony with the feet movements landing on the perch (Ota et al. 2017). The sound data from the normal-speed video was digitized using Raven Pro 1.5 (Bioacoustic Research Program 2017) at a sampling frequency of 44.1 kHz and 16-bit resolution. I did not use high-speed video data for the acoustic analysis since high-speed recording cannot record sounds.

To gain insights into the role and functions of multimodal signals, I checked the environmental conditions (i.e., if the bird was on the branch of a tree or on the ground), the presence and the sex of a signal receiver on the same substrate during courtship based on the video recordings. I also examined if the courtship display was a solo or duet (i.e., two birds coordinating their courtship display with a degree of temporal precision; Hall 2004).

Supplementary results

Including the observations without video recordings, I observed the dance display of blue-capped cordon-bleus 49 times (49 males) and red-cheeked cordon-bleus 15 times (11 males and four females). While there were both blue-capped and red-cheeked cordon-bleus in both the Mbeya and Iringa regions, I detected more blue-capped cordon-bleus in Mbeya region and more red-cheeked cordon-bleus in Iringa region (personal observation). Forty-eight courtship displays of male blue-capped cordon-bleus were observed in the Mbeya region, and one courtship display of a male blue-capped cordon-bleu and all 15 courtship displays of red-cheeked cordon-bleus were observed in the Iringa region. Although I observed multiple nests used by blue-capped and red-cheeked cordon-bleus in the field site (Ota in prep), I did not observe that cordon-bleus perform courtship displays around the nest.

During courtship, cordon-bleus held pieces of grass, pieces of the leaves of *Acacia* or *Faidherbia* spp., bird feathers, flowers (*Mollugo nudicaulis*), or nothing (Fig. S2). Cordon-bleus seemed to use several different species of grasses, but I could not identify them because of the video resolution and the dry condition of the grasses.

References

Altmann J (1974). Observational study of behavior: sampling methods. Behaviour, 49:227-266. https://doi.org/10.1163/156853974X00534

Baker NE, Baker EM. (2016) Tanzania bird atlas: Distribution and seasonality. http://www.tanzaniabirdatlas.net. Accessed 1 Oct 2018

Bioacoustic Research Program (2017) Raven Pro: interactive sound analysis software (version 1.5). The Cornel Lab of Ornithology, Ithaca http://www.birds.cornell.edu/raven.

Goodwin D (1982) Estrildid finches of the world. Cornell University Press, Ithaca

Hall ML (2004) A review of hypotheses for the functions of avian duetting. Behav Ecol Sociobiol 55:415–430. https://doi.org/10.1007/s00265-003-0741-x

Ota N, Gahr M, Soma M (2015) Tap dancing birds: the multimodal mutual courtship display of males and females in a socially monogamous songbird. Sci Rep 5:16614. https://doi.org/10.1038/srep16614

Ota N (2017) Complex sexual signals in the mutual courtship display of blue waxbills. Dissertation, Hokkaido University

Ota N, Gahr M, Soma M (2017) Songbird tap dancing produces non-vocal sounds. Bioacoustics 26:161–168. https://doi.org/10.1080/09524622.2016.1231080

Soma M (2018). Sexual selection in Estrildid finches, with further review of the evolution of nesting material holding display in relation to cooperative parental nesting. Jpn J Anim Psychol 68:121–130. https://doi.org/10.2502/janip.68.2.2

Supplementary figures

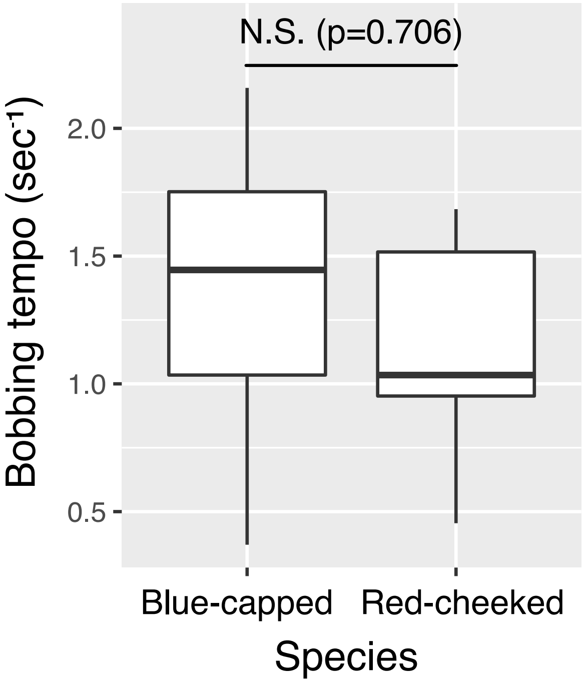

Fig. S1 The comparison of bobbing tempo (sec^{-1}) between male blue-capped (*Uraeginthus cyanocephalus*) and male red-cheeked cordon-bleus (*Uraeginthus bengalus*). Box plots show medians and quartiles and the whiskers include the range of values within 1.5 times the interquartile range.

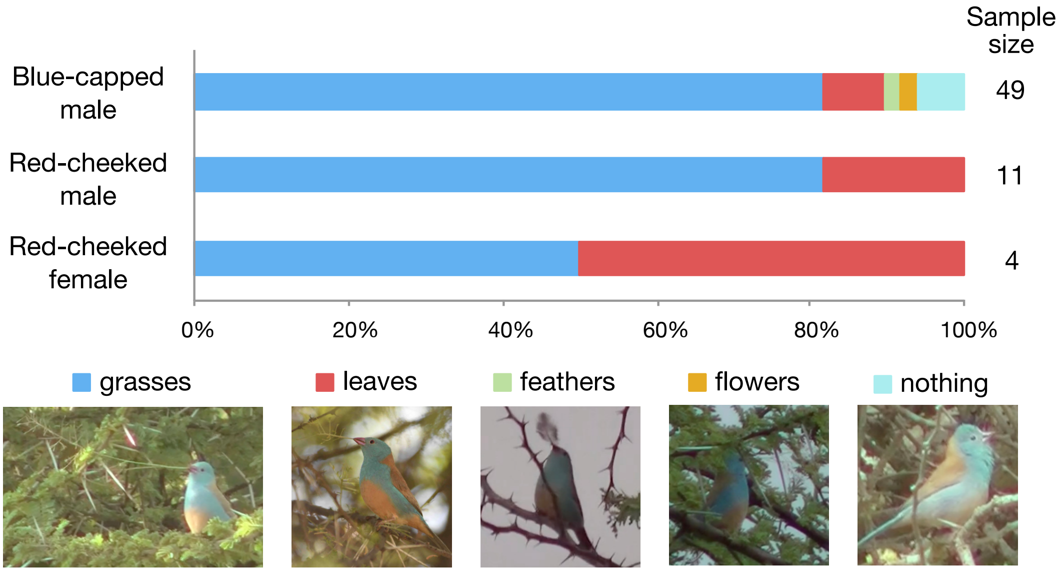

Fig. S2 The proportion of items held in the beak during courtship in male blue-capped cordon-bleus (*Uraeginthus cyanocephalus*) and male and female red-cheeked cordon-bleus (*Uraeginthus bengalus*). Numbers at the right of bar graphs represent sample size used for calculating the proportion. Different items are indicated by different colors in the bar graphs. The categorization of items and the assignments of colors are shown with photos under the graph bars.

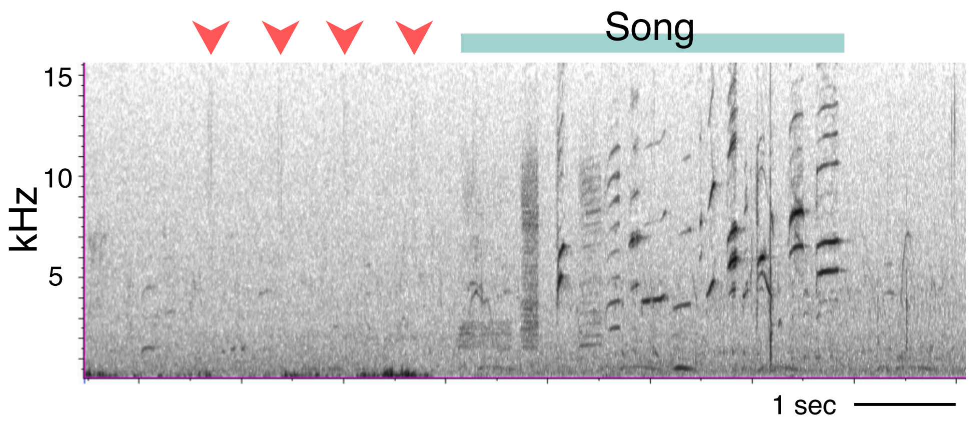

Fig. S3 A spectrogram of non-vocal sounds (red arrows) produced by dancing followed by a song in a male blue-capped cordon-bleu (*Uraeginthus cyanocephalus*; settings: brightness 50, contrast 50, and spectrogram window size 512); blotted is the frequency (kHz) against the time (sec).

Supplementary table

Table S1 A result of a linear mixed model, testing species differences between blue-capped (*Uraeginthus cyanocephalus*) and red-cheeked cordon-bleus (*Uraeginthus bengalus*) in bobbing tempo. Bird ID was included as a random effect in a model. Estimated values for effects that contained a "species" term are for red-cheeked cordon-bleus.

Response variable	Fixed effect	Coefficient	SE	t-value	P-value	Distribution
Bobbing tempo	Species	-0.072	0.190	-0.382	0.706	Gaussian

Supplementary video

Video 1. A normal-speed movie of a male blue-capped cordon-bleu (*Uraeginthus cyanocephalus*) courtship display and a high-speed movie of male blue-capped cordon-bleu performing a dance display near a female blue-capped cordon-bleu.
